# Prior Experience with Food Reward Influences the Behavioral Responses of the Honeybee *Apis mellifera* and the Bumblebee *Bombus lantschouensis* to Tomato Floral Scent

**DOI:** 10.3390/insects11120884

**Published:** 2020-12-14

**Authors:** Hong Zhang, Shuang Shan, Shaohua Gu, Xinzheng Huang, Zibo Li, Adel Khashaveh, Yongjun Zhang

**Affiliations:** 1 State Key Laboratory for Biology of Plant Diseases and Insect Pests, Institute of Plant Protection, Chinese Academy of Agricultural Sciences, Beijing 100193, China; zhanghong@caas.cn (H.Z.); shanshuang@caas.cn (S.S.); 15733226110@163.com (Z.L.); akhashaveh@caas.cn (A.K.); 2 Key Laboratory for Insect-Pollinator Biology of the Ministry of Agriculture and Rural Affairs, Institute of Apicultural Research, Chinese Academy of Agricultural Sciences, Beijing 100093, China; 3 Department of Entomology, College of Plant Protection, China Agricultural University, Beijing 100193, China; gushaohua@cau.edu.cn (S.G.); huangxinzheng@cau.edu.cn (X.H.)

**Keywords:** honeybee, bumblebee, tomato floral scent, pollination, prior experience, behavior preference

## Abstract

**Simple Summary:**

Bees are important pollinators for many agricultural crops. Compared with bumblebees, honeybees are less attracted to tomato flowers. Floral scent usually plays an important role in mediating the foraging behavior of bees, and tomato flowers release special scents. However, little is known about how tomato floral scent regulates the foraging behaviors of these two bee taxa. In the current study, we investigated the foraging behaviors of the widely used pollinator honeybee *Apis mellifera* and a native bumblebee, *Bombus lantschouensis*, on tomato flowers to evaluate the potential application of these two bee species for tomato pollination in solar greenhouses. Moreover, we determined whether honeybees and bumblebees show different responses to tomato floral scent and how innate biases and prior experience influence bee choice behavior. We found that naïve bees showed no preference for tomato floral scent but could develop such a preference after learning to associate tomato floral scent with a food reward on the basis of foraging experience or scent-learning procedures. We conclude that scent-learning experiences with food reward can change the innate bias of bees and could be utilized to improve the pollination service efficiency of bees for commercial crops.

**Abstract:**

Bee responses to floral scent are usually influenced by both innate biases and prior experience. Honeybees are less attracted than bumblebees to tomato flowers. However, little is known about how tomato floral scent regulates the foraging behaviors of honeybees and bumblebees. In this study, the foraging behaviors of the honeybee *Apis mellifera* and the bumblebee *Bombus lantschouensis* on tomato flowers in greenhouses were investigated. Whether the two bee species exhibit different responses to tomato floral scent and how innate biases and prior experience influence bee choice behavior were examined. In the greenhouses, honeybees failed to collect pollen from tomato flowers, and their foraging activities decreased significantly over days. Additionally, neither naïve honeybees nor naïve bumblebees showed a preference for tomato floral scent in a Y-tube olfactometer. However, foraging experience in the tomato greenhouses helped bumblebees develop a strong preference for the scent, whereas honeybees with foraging experience continued to show aversion to tomato floral scent. After learning to associate tomato floral scent with a sugar reward in proboscis extension response (PER) assays, both bee species exhibited a preference for tomato floral scent in Y-tube olfactometers. The findings indicated that prior experience with a food reward strongly influenced bee preference for tomato floral scent.

## 1. Introduction

Tomato (*Lycopersicum esculentum*) is one of the most consumed vegetables worldwide. China has the largest planted area of tomato and is the largest tomato producer in the world [1]. Protected cultivation (e.g., in tunnels, solar greenhouses, and multispan greenhouses), which helps bridge the seasonal gap in tomato production, has become an important cultivation system and accounts for 57.2% of the total tomato production in China [2]. Unlike traditional greenhouses, which require significant energy consumption and high capital, solar greenhouses use solar radiation for heating and depend on top and bottom vents to regulate temperature and humidity. Solar greenhouse cultivation has emerged as one of the most popular tomato cultivation systems in northern China [3,4,5].

Bumblebee pollination plays an important role in protected tomato cultivation. In tomato flowers, stamens form a “cone” unit surrounding the pistil, and anthers dehisce longitudinally towards the flower center [6]. Although tomato is self-compatible, vibration is necessary to facilitate the release of pollen from anthers and its deposition on stigmas [7]. Bumblebees have the ability to contract their flight muscles and vibrate tomato anthers to release pollen grains; this behavior is called ‘buzz pollination’ [8,9]. Because of their buzz-pollination ability, bumblebees have been successfully used for tomato pollination. The best-known bumblebee species, *Bombus terrestris*, which originated in Europe, has been exported worldwide for tomato pollination [10]. In China, imported commercial *B. terrestris* colonies have played a primary role in the bee pollination market for protected-cultivation tomato. Although pollination by *B. terrestris* has provided a great deal of economic value to tomato growers, the introduction of *B. terrestris* to nonnative areas has led to biological invasion, which has resulted in the extinction or population declines of local bumblebee species [11,12,13]. Therefore, the selection and utilization of local bumblebee species or other bee species for tomato pollination are drawing attention [14,15,16]. China has the greatest bumblebee diversity in the world [17]. Among the local bumblebee species in China, *B. lantschouensis* shows the greatest potential for commercial pollination because of its large colony size and good adaptability to solar greenhouses [18,19]. However, in the literature on *B. lantschouensis*, many studies have focused only on molecular biology, and research on the pollination biology of *B. lantschouensis* in tomato is rare.

In addition to bumblebees, the honeybee *Apis mellifera*, which is the most widely used pollinator species in the world, can pollinate tomatoes. Although honeybees do not have the ability to sonicate tomato flowers, they can drum against the anther tips with their forelegs or milk the anthers to release pollen [20]. In some studies, honeybee pollination has been shown to improve tomato yield and quality [21,22,23]. However, others have found that honeybee pollination of tomato is not effective [24,25]. Bispo dos Santos et al. found that several honeybee individuals visited tomato flowers in open fields and that tomato fruits in greenhouses with honeybee colonies presented the same size and weight as those in greenhouses with no bee pollinators [26]. Although several studies have investigated the pollination behavior or efficiency of honeybees for tomatoes, few have aimed to understand the factors that could explain why honeybees are unreliable pollinators.

Plant floral scent plays crucial roles in mediating the foraging behavior of insect pollinators [27]. Generally, pollinator behavioral responses to floral scent are dependent on both innate bias and learned experience [28]. Bees show innate species-specific preferences for (or aversion to) floral scent. The foraging preference of *B. impatiens* for the scent of *Mimulus guttatus* has been shown to be driven by olfactory cues that are innately attractive to the bee [29]. The innate responses to floral scent might vary among bee species. Naïve bumblebees were found to be attracted to aromatic-dominated floral scent from the Asteraceae species *Cirsium arvense*, whereas naïve honeybees were repelled by the terpenoid-dominated floral scent of another Asteraceae plant, *Achillea millefolium* [30]. In addition, floral scent acts as a learning cue that enables bees to identify flowers containing a reward [31,32,33]. Bumblebees may develop a preference for synthetic volatiles after they obtain a reward from artificial flowers containing these compounds [34].

It has been reported that volatile organic compounds from tomato plants can influence the foraging behavior of herbivore pests [35,36,37]. However, few studies have focused on the influences of tomato floral scent on bee pollinator foraging behavior. A negative relationship has been found between the daily release dynamics of β-phellandrene and (+)-2-carene from tomato flowers and the daily foraging activity of bumblebees, suggesting that bumblebees might prefer to visit tomato flowers containing less of the two compounds [38]. However, both the emission dynamics of tomato floral scent and the daily foraging activities of bumblebees are highly dependent on time of day and environmental temperature. It is difficult to conclusively determine the repellent effects of these two compounds on bumblebee foraging behavior. Moreover, whether tomato floral scent differentially influences the foraging behaviors of bumblebees and honeybees and is thus responsible for the different pollination efficiencies of these two bee taxa in tomato is still unknown.

In the present study, we investigated the foraging behaviors of the widely used pollinator honeybee *A. mellifera* and the native bumblebee *B. lantschouensis* on tomato flowers to evaluate their potential as tomato pollinator in greenhouses. We determined how tomato floral scent mediated the behavioral responses of honeybees and bumblebees and whether differences existed between the two bee species. Our findings provide valuable insights into pollinator–plant interactions on tomato from the perspective of insect chemical ecology and improve our utilization of bee pollinators for tomato pollination in solar greenhouses.

## 2. Materials and Methods

### 2.1. Plants and Bees

**Figure 1:**
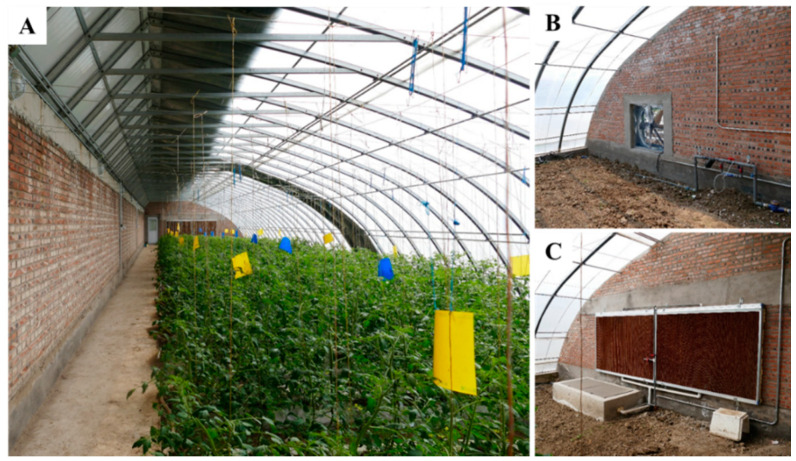
A solar greenhouse and the cooling devices used in the hot summer. (**A**), tomato plants and sticky card traps in a greenhouse; (**B**), cooling fan; (**C**), water curtain.

Tomatoes (*Lycopersicum esculentum* ‘Provence’) were planted in six solar greenhouses located in Beijing, China. The six greenhouses were of the same type and subjected to the same horticultural management. The area of each greenhouse was 525 m^2^ (7 × 75 m). The back and side walls of the greenhouses were composed of brick. An aisle of 0.5 m in width was maintained along the back wall. The sunny side was covered with two transparent and overlapping polyethylene film layers on the top of the greenhouse, and one of the layers could be opened and closed. Each greenhouse was equipped with fly netting to prevent bees from flying out. Yellow and blue sticky card traps were used to control insect pests in the greenhouse. On the side walls, fans and a water curtain were installed to reduce the air temperature during the hot summer (Figure 1). Tomato plants were transplanted to the greenhouse in 100 rows of 12–14 plants each, with a spacing of 0.7 m between rows. All rows were perpendicular to the back wall, with a length of 6.5 m from north to south. Five inflorescences were retained on each plant to produce fruit. The inflorescences bloom sequentially from the bottom to the top of the plants. The peak blooming period of each inflorescence usually lasts 3–4 days, and the blooming periods of adjacent inflorescences overlap slightly. In this study, the whole flowering period of the tomato plants lasted for ca. 25–30 days. During the flowering period, the temperature in the greenhouses was maintained within 15–32 °C, and the relative humidity (RH) was maintained within 30–65%, conditions favorable for bee flight.

The colonies of honeybees and bumblebees used in the study were provided by the Institute of Apicultural Research, Chinese Academy of Agricultural Sciences. The six greenhouses were randomly assigned to honeybee or bumblebee colony, with tomato flowers in three of the six greenhouses being pollinated by honeybees, and those in the other three being pollinated by bumblebees. The foraging behavior of bees on tomato flowers was investigated from 25 April to 5 June, 2018. At the beginning of the tomato flower blooming period, three *A. mellifera* colonies, each consisting of three frames of approximately 6000 workers, and three *B. lantschouensis* colonies, each consisting of approximately 60 workers, were placed in each greenhouse. Both the honeybee and bumblebee colonies were in healthy condition, and each colony had a queen and broods in their hives. Because tomato flowers are nectar free, each honeybee colony was provided a frame of covered honey, and each bumblebee colony was provided two kilograms of sugar syrup (50% sugar content, *w*/*w*).

### 2.2. Foraging Behaviors of Bees in Solar Greenhouses

The foraging behavior of bees was investigated during the peak blooming period of each inflorescence every 5–6 days after the first inflorescence bloomed. A total of four days of data were recorded in the peak blooming period of each of the first four inflorescences during the whole flowering period. The entry and exit behaviors of bee foragers at the hive entrance within the first 30 min of every hour between 05:00 and 18:00 were monitored by a digital camera (Yi-2, YI Technology, Shanghai, China). Pollen-foraging behavior was recorded by monitoring the returning pollen foragers at the hive entrance, which pollen foragers were identified based on the presence of pollen in their corbiculae. The proportion of pollen foragers was used to compare the pollen-foraging behaviors between honeybees and bumblebees [19]. The bee visitor density was measured by scan sampling 100 opening tomato flowers in each of three plots from each greenhouse [39]. The three sampling plots each contained two or three adjacent rows and were distributed evenly in the greenhouse. Sampling was performed by walking slowly between the set rows, and a bee visitor was recorded if a bee was present at a given flower that was being seen for the first time. The bee visitor density was recorded every hour from 08:00 to 16:00 on each of the four days.

### 2.3. Floral Scent Collection

The tomato floral scent was collected using a dynamic headspace extraction device [40]. Prior to flowering, individual buds were isolated in fabric mesh bags (mesh diameter: 2 mm) to prevent visits by bees. Once the flowers opened in the next morning, they were placed in a polyethylene plastic cup (upper diameter: 490 mm, bottom diameter: 370 mm, height: 35 mm, volume: 35 mL) to collect scent. The flowers in the cups opened normally and were not jostled. Each cup contained an inlet and an outlet to let air in and out. The inlet was made in the middle of the lid of the cup. A vacuum pump (QC-1B, Beijing Institute of Labor Instrument, Beijing, China) was utilized to pull fragrant headspace air through sorbent traps at a flow rate of 1.5 mL/min. Air purified via an activated charcoal column (100 mm length × 5 mm diameter) was pumped through the cup, and volatiles were forced into a glass trap (120 mm length × 8 mm diameter) containing 50 mg Porapak Q (80/100 mesh, Supelco, Bellefonte, PA, USA). All of the connections were made with Teflon tape. The collection of volatiles was conducted over 6 h from 09:00 to 15:00. Then, the glass traps were eluted twice with a total of 300 μL of HPLC grade hexane (Sigma-Aldrich, Oakville, ON, Canada), and the volatile compounds were stored in sample vials (2 mL; Agilent Technologies, California, USA) at −80 °C until use.

### 2.4. Behavioral Responses of Bees to Tomato Floral Scent

To investigate the innate behavioral responses of bees to tomato floral scent and whether prior foraging experience or reward-based learning experience affects the choice behavior of bees, Y-tube olfactometer assays were performed indoors. Three treatment groups of bees were established: (1) an innate scent bias group, comprising bees from colonies that had never been used to pollinate tomato planted in a greenhouse or outside (type 1); (2) a prior foraging experience group, comprising bees from colonies in tomato greenhouses (type 2); and (3) a reward-learned experience group, comprising bees that (a) were from colonies that had never been used to pollinate tomato planted in greenhouse or outside and (b) had been trained to associate tomato floral scent with a high-quality reward via the proboscis extension response (PER) (type 3). For the bees of type 1 and type 3, a total of 113 honeybee individuals were sampled from three honeybee colonies, whereas 120 bumblebee individuals were sampled from 10 bumblebee colonies. The bumblebee colonies were fed sugar syrup (50% *w*/*w*) and pollen paste (a mixture of fresh oilseed rape pollen and apricot pollen collected by honeybee colonies) every other day under conditions of 26 ± 1 °C and 65 ± 3% RH [18]. Bees were individually placed in ventilated plastic tubes and then randomly assigned to two groups, type 1 and type 3. The bees of type 1 were directly tested in a Y-tube olfactometer, whereas bees of type 3 were trained in PER assays and then tested in a Y-tube olfactometer (The details of the Y-tube and PER assays are presented in Section 2.4.1 and Section 2.4.2). For the bees of type 2, we caught foragers from colonies in the six tomato greenhouses. The bees were caught one week after the hives were placed in the tomato greenhouses. For the bumblebees, a total of 52 pollen foragers that returned to the hives were individually caught at the hive entrance. For the honeybees, we failed to find pollen foragers in the tomato greenhouses, so a total of 43 honeybee workers that returned to the hive entrance were sampled. Bees caught in the tomato greenhouses were individually placed in plastic tubes and then tested in a Y-tube olfactometer within 6 h. The details are as follows.

#### 2.4.1. Dual-Choice Behavior Trials

Dual-choice tests were performed in a glass Y-tube olfactometer to investigate the behavioral responses of bees to tomato floral scent [41]. The olfactometer was composed of 40 mm-diameter glass tubing and consisted of a 200 mm-long central tube and two 150 mm-long lateral arms; the angle between the two lateral arms was 60°. The olfactometer was placed within a 100 × 100 × 60 cm observation chamber that was illuminated with a 5 W red LED light bulb and maintained at 25 ± 1 °C. A vacuum pressure pump (QC-1B, Beijing Institute of Labor Instrument, Beijing, China) forced room air through activated charcoal and an Erlenmeyer flask filled with distilled water. Airflow through each of the olfactometer arms was kept at 300 mL/min and entered the apparatus through Teflon tubing. The 10 μL of tested chemicals was applied to a 20 × 5 mm filter paper strip and immediately placed into a flask of one liter. In these assays, each bee was offered a choice between 10 μL of floral scent and 10 μL of hexane. The bees were individually released at the base of the central arm of the Y-tube and observed for 5 min. If a bee did not make a choice after this period, it was removed and recorded as showing no choice. Bees that walked into one of the terminal arms and remained there for at least 5 s or traveled two-thirds the length of a lateral arm were recorded as having chosen the odor offered through that arm. Each bee was tested only once. After testing a total of five bees, the positions of the arms containing the treatment and control odors were reversed to avoid positional bias. The olfactometer was replaced with a clean one after 10 individuals had been tested.

#### 2.4.2. PER Assays

A total of 50 naïve honeybees and 45 naïve bumblebees were trained for PER assays [42,43]. Honeybees were harnessed in a 0.5 mL centrifuge tube, whereas bumblebees were harnessed in a 2 mL centrifuge tube. The bottom of the centrifuge tube was cut off according to the head size of the bees, and it was ensured that their heads and proboscises could move freely. A plastic straw was cut to a suitable length and used to support the bee body inside the centrifuge tube. The bees were then fed a sugar solution (50% *w*/*w*) to satiation and isolated in the dark for 8 h (at 20 °C and an RH of 50%). Before the test, bees that failed to show a response to the sucrose solution or showed unhealthy conditions were discarded. Two vacuum pumps (QC-1B, Beijing Institute of Labor Instrument, Beijing, China) were used to generate airflow. Odors were delivered to the bees via 1000 mL pipette tips connected through Teflon tubes to vacuum pumps. One of the tips contained filter paper treated with 10 μL of hexane, and the other contained filter paper treated with 10 μL of tomato floral scent. There were two stimulus treatments: (1) conditioned stimulus (CS) treatment, wherein bees were exposed to a flow containing floral odor; and (2) unconditioned stimulus (US) treatment, wherein the bee antennae were touched with a toothpick dipped in a 50% sugar solution, and the bees were allowed to lick the toothpick. Briefly, in each trial, the bee was exposed to a constant flow of hexane for 15 s and then to CS for 3 s, followed by 3 s of exposure to CS and US together. Subsequently, the bee was allowed to lick the sucrose solution from the toothpick for 1 s. Finally, the bee was again exposed to a hexane flow for 8 s. Each bee was trained sequentially in 10 trials, with an intertrial interval of 10 min. To determine whether the bees remembered the learned association, memory retention tests were conducted at 1 h, 2 h and 24 h after the acquisition phase by providing only the floral odor. A bee that responded with the extension of the proboscis during the first 3 s of CS exposure was scored as 1, whereas a bee that responded only to the sugar reward or did not respond at all was scored as 0.

### 2.5. Statistical Analysis

All data were analyzed using IBM SPSS 20 (Chicago, IL, USA). First, we investigated whether the foraging behaviors in tomato solar greenhouses differed between honeybees and bumblebees. General linear repeated measure models were used, with the ‘number of outgoing foragers’, ‘proportion of pollen foragers’ and ‘bee visitor density’ as the response variables; ‘time’ (with the four sampling days during the tomato flowering period as the levels) as the within-subject factor; and ‘bee species’ and ‘greenhouse’ as between-subject factors. For ‘bee visitor density’, to investigate whether bee visitors were distributed evenly in the tomato greenhouses, ‘plot’ (with the three sampling plots in each greenhouse as the levels), ‘bee species’ and ‘greenhouse’ were set as between-subject factors. The effects of ‘greenhouse’ on ‘number of outgoing foragers’ (F_4.49_ = 0.165, *p* = 0.995), ‘proportion of pollen foragers’ (F_4.49_ = 1.627, *p* = 0.182), and ‘bee visitor density’ (F_4,144_ = 1.200, *p* = 0.313) and the effect of ‘plot’ on ‘bee visitor density’ (F_2,144_ = 0.076, *p* = 0.926) were not significant; hence, the between-subject factors ‘greenhouse’ and ‘plot’ were removed from the final models. Mauchly’s test of sphericity indicated that the assumption of sphericity was violated for ‘number of outgoing foragers’ (*p* < 0.000), ‘proportion of pollen foragers’ (*p* = 0.026), and ‘bee visitor density’ (*p* < 0.000); hence, Greenhouse-Geisser corrections were used (‘number of outgoing foragers’: ε = 0.780; ‘proportion of pollen foragers’: ε = 0.847; ‘bee visitor density’: ε = 0.869). As most of the foraging behavior data were not normally distributed, as determined by Shapiro–Wilk normality test, nonparametric Mann–Whitney U tests were used to compare the foraging behaviors of the two bee species on each sampling day. Moreover, linear regression models were used to analyze the effect of pollination period (with the four sampling days during the different tomato flowering periods as the levels) on the foraging activity of the honeybees and bumblebees in the tomato greenhouses.

The preferences of the honeybees and bumblebees used in the dual-choice behavior trials in the Y-tube olfactometer were analyzed with chi-square tests. A one-sample chi-square test was used to analyze whether significant differences existed in the choice of the bees between tomato floral scent and hexane. The Pearson chi-square test was used to evaluate the impacts of foraging experience and learned experience on the choice behavior of the bees and to compare the differences in choice behavior between the two bee species.

Generalized linear mixed models (GLMMs) were used to analyze the learning performance and memory retention of the honeybees and bumblebees in response to tomato floral scent with PER as the response factor (bee exhibiting PER = 1, bee not exhibiting PER = 0) and trial/time point, bee species and their two-way interaction as fixed factors. Because there was no significant interaction effect between bee species and trial/time point during the learning trials (GLMM: F_9,800_ = 0.099, *p* = 1.000) and memory retention (GLMM: F_2,240_ = 0.869, *p* = 0.421), the two-way interaction was removed from the fixed factors in the final models. Additionally, binary logistic regression with a logit link function was utilized in the model.

## 3. Results

### 3.1. Pollination Behavior of Honeybees and Bumblebees in Tomato Greenhouses

**Figure 2:**
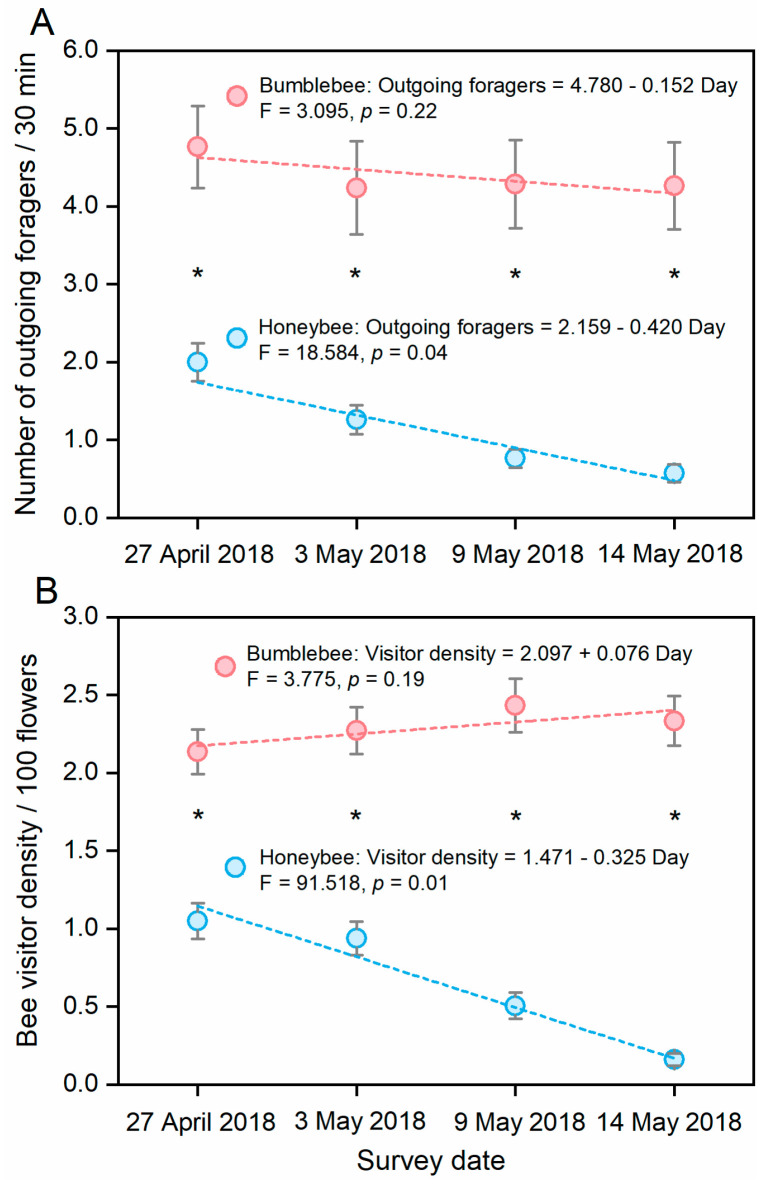
Daily pattern of (**A**) the number of outgoing foragers and (**B**) visitor density of *Apis mellifera* and *Bombus lantschouensis* in tomato greenhouses on different sampling dates. The behavior data were obtained in six greenhouses during the tomato flowering period, and a total of 168 records of the ‘number of outgoing foragers’ and a total of 324 records of ‘bee visitor density’ were obtained for both honeybees and bumblebees. Data are presented as the mean ± S.E. Asterisks indicate significant differences (*p* < 0.05) between *A. mellifera* and *B. lantschouensis* on each sampling day based on nonparametric Mann-Whitney U methods. Dashed lines depict linear regressions based on the mean value for each sampling date.

We obtained a total of 168 video records of entry and exit behavior and 324 manual records of visitor density for each bee species in the six greenhouses. Honeybees and bumblebees showed significant differences in the numbers of outgoing foragers (Figure 2A, time × bee species interaction: F_2.340, 124.041_ = 4.042, *p* = 0.015) and flower visitors (Figure 2B, time × bee species interaction: F_2.606, 416.955_ = 18.648, *p* < 0.000) during different tomato flowering periods. On each sampling day, significantly fewer honeybee foragers than bumblebee foragers were observed exiting the hives (Mann–Whitney U test: day 1: U = 473.00, *p* < 0.000; day 2: U = 476.00, *p* < 0.000; day 3: U = 250.00, *p* < 0.000; day 4: U = 213.00, *p* < 0.000) and visiting tomato flowers (Mann–Whitney U test: day 1: U = 1766.50, *p* < 0.000; day 2: U = 1473.50, *p* < 0.000; day 3: U = 806.00, *p* < 0.000; day 4: U = 331.00, *p* < 0.000) in tomato greenhouses. Furthermore, the number of honeybee foragers exiting hives (F_1,3_ = 18.58, *p* = 0.04) and visiting flowers (F_1,3_ = 91.518, *p* = 0.01) decreased significantly over the study days in the tomato greenhouses. On the last sampling day, almost no honeybee foragers were observed visiting flowers in tomato greenhouses. However, there was no difference in bumblebee foraging behavior among sampling days (number of outgoing foragers: F_1,3_ = 3.095, *p* = 0.22; bee visitor density: F_1,3_ = 3.775, *p* = 0.19).

**Figure 3:**
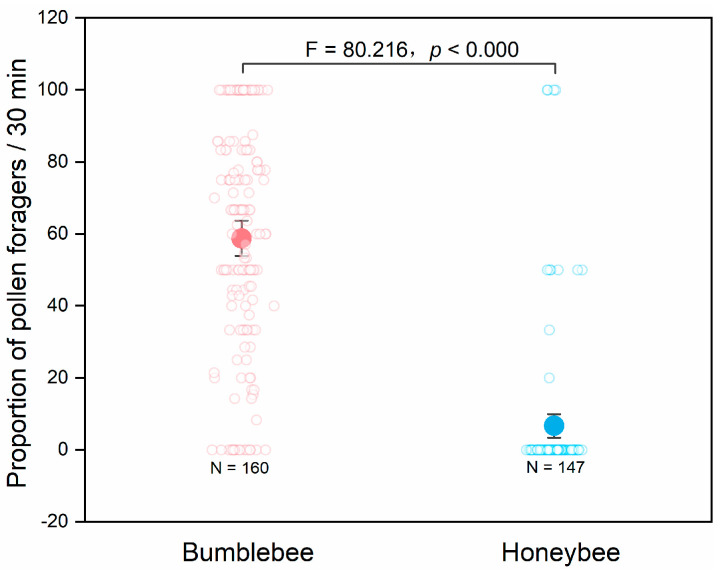
The proportions of pollen foragers of *Apis mellifera* and *Bombus lantschouensis* at the hive entrance in tomato greenhouses. Behavior data were obtained in six greenhouses during the tomato flowering period, and a total of 160 and 147 records were obtained for honeybees and bumblebees, respectively. Data are presented as the mean ± 95% confidence interval. A general linear repeated measure model was used to compare the proportion of pollen foragers between the two bee species.

A total of 12 of 207 honeybee foragers and a total of 477 of 851 bumblebee foragers carried pollen back to the hive. Although the honeybees had a much larger colony size than the bumblebees, significantly fewer honeybee foragers than bumblebee foragers were able to obtain pollen as a food reward when visiting tomato flowers (Figure 3, F_1,53_ = 80.216, *p* < 0.000). The pollen-collecting behaviors of the bees were not affected by the flowering period of tomato (F_2.542,134.740_ = 0.962, *p* = 0.402).

### 3.2. Influence of Foraging Experience on Bee Response to Tomato Floral Scent

**Figure 4:**
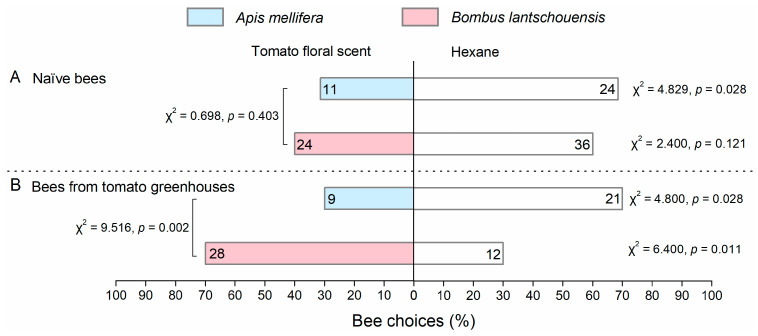
Behavioral responses of *Apis mellifera* and *Bombus lantschouensis* to tomato floral scent in Y-tube olfactometer. (**A**) Naïve bees from colonies that had never been used to pollinate tomato planted in a greenhouse or outside; (**B**) Bees from colonies in tomato greenhouses. The numbers in each bar represent the numbers of bees that made a choice. A one-sample chi-square test was used to analyze whether significant differences existed in bee choices between tomato floral scent and hexane. Pearson’s chi-square test was used to analyze the influence of foraging experience on the choice of the bees and to compare the differences in choice between the two bee species.

Foraging experience in tomato greenhouses showed a significant influence on the choice behavior of bumblebees (Pearson chi-square test: χ^2^ = 8.654, df = 1, *p* = 0.003) but had no effect on that of honeybees (Pearson chi-square test: χ^2^ = 0.004, df = 1, *p* = 0.952). For bees without foraging experience in tomato greenhouses, no significant difference in choice behavior toward tomato floral scent was found between the honeybees and bumblebees (Figure 4A, Pearson chi-square test: χ^2^ = 0.698, df = 1, *p* = 0.403). Both bee species showed aversion to tomato floral scent, and naïve honeybees showed significant aversion (Figure 4A, one-sample chi-square test: χ^2^ = 4.829, df = 1, *p* = 0.028). Bumblebees and honeybees collected from tomato greenhouses showed significantly different responses to floral scent (Figure 4B, Pearson chi-square test: χ^2^ = 9.516, df = 1, *p* = 0.002). Bumblebees with foraging experience were more attracted to tomato floral scent than to hexane (Figure 4B, one-sample chi-square test: χ^2^ = 4.829, df = 1, *p* = 0.028), whereas honeybees still showed aversion to tomato floral scent (Figure 4B, one-sample chi-square test: χ^2^ = 4.800, df = 1, *p* = 0.028).

### 3.3. Influence of Learned Experience on Bee Response to Tomato Floral Scent

**Figure 5:**
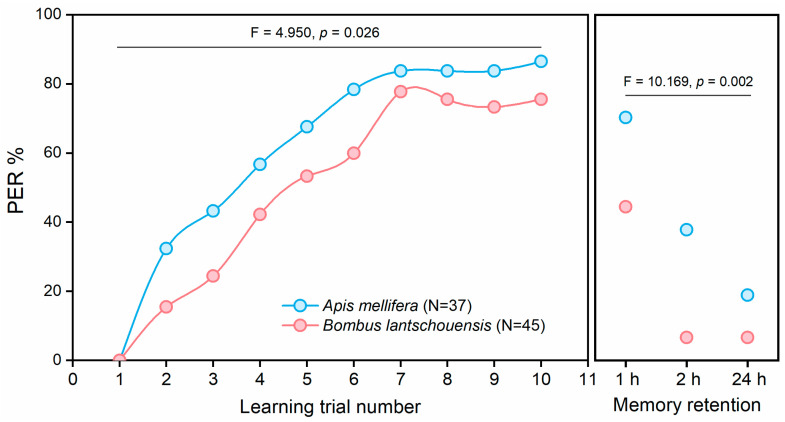
Proboscis extension responses (PERs) toward tomato floral scent in *Apis mellifera* and *Bombus lantschouensis*. Dots represent the proportions of bees showing a PER to tomato floral scent in each trial. The box on the right shows the memory retention of the bees 1, 2 or 24 h after training. Generalized linear mixed models were used to analyze the learning performance and memory retention of honeybees and bumblebees in response to tomato floral scent.

**Figure 6:**
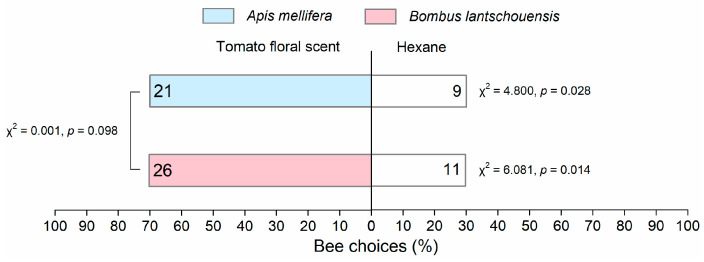
Behavioral responses of *Apis mellifera* and *Bombus lantschouensis* toward tomato floral scent in Y-tubes after learned experience associating tomato floral odors with a high-quality reward via the proboscis extension response (PER). The numbers in each bar represent the numbers of bees that made a choice. A one-sample chi-square test was used to analyze whether significant differences existed in bee choice between tomato floral scent and hexane. The Pearson chi-square test was used to analyze the influence of learned experience on bee choice for tomato floral scent and to compare the differences in choice behavior between honeybees and bumblebees.

The learned experience of associating scent with a food reward influenced the responses of both honeybees (Pearson chi-square test: χ^2^ = 9.615, df = 1, *p* = 0.002) and bumblebees (Pearson chi-square test: χ^2^ = 8.396, df = 1, *p* = 0.004) to tomato floral scent. In PER assays, honeybees and bumblebees were able to successfully associate tomato floral scent with a food reward (Figure 5). After learning, both bee species showed a strong preference for the tomato floral scent in Y-tube olfactometer, and this preference did not differ between the two species (Figure 6).

No bee extended its proboscis toward the tomato floral scent in the initial learning trial. During the learning trials, both honeybees and bumblebees were able to associate the tomato floral scent with a reward, and the number of bees extending their proboscises toward the tomato floral scent increased significantly (GLMM: F_9,800_ = 15.632, *p* = 0.000). Compared with bumblebees, honeybees showed significantly higher learning scores during the learning trials (GLMM: F_1,800_ = 4.950, *p* = 0.026) and significantly greater memory retention (GLMM: F _1,240_ = 10.169, *p* = 0.002).

## 4. Discussion

### 4.1. Bee Foraging Behaviors in Tomato Greenhouses

The native bumblebee *B. lantschouensis* was well adapted to the solar greenhouse environment in this study and collected pollen from tomato flowers efficiently, indicating its large potential for tomato pollination in greenhouses. Although honeybees present a huge advantage over bumblebees because of their larger colony size, significantly fewer honeybee foragers were observed working on tomato flowers in the current study. The microclimate of solar greenhouses (e.g., solar radiation, temperature, RH, and airflow) may influence bee foraging behaviors [44]. According to a previous study, honeybees can adapt well to the solar greenhouse environment and are able to collect enough pollen from peach [19]. We speculate that the environmental conditions were not the cause of the lower foraging activity of honeybees in the tomato solar greenhouses.

In our study, the foraging activity of honeybees decreased significantly over the days after being placed in the tomato greenhouse (Figure 2). Gemeda et al. observed a similar phenomenon in pear: the pollen-foraging activity of the honeybee *A. mellifera* on pear flowers decreased steadily over the study days [45]. In their study, despite the high flower density and abundant pollen of pear flowers, *A. mellifera* showed a significantly greater preference for pollen from other flowering plants. It has been reported that compared with tomato flowers, honeybees are much more easily attracted to other flowers [46]. Our study was conducted in isolated greenhouses, where tomato flowers were the monofloral pollen source. In the absence of other flowering plants, the honeybees exhibited low outgoing activity (Figure 2A). Broods and larvae can stimulate honeybee pollen-foraging behavior, which can increase foraging activity [47]. However, in a previous study of tomato pollination in a greenhouse, no significant differences in foraging activity between ‘brood’ and ‘no-brood’ honeybee colonies were observed [23]. In our study, the pollination colonies of both honeybees and bumblebees contained broods when they were placed in the tomato greenhouses. However, the presence of the broods did not appear to stimulate honeybee foraging activity on tomato flowers. However, we did not investigate differences in the ratio of open brood between the two bee species. The open brood stage should be considered when comparing the foraging behaviors of different bee pollinators in future studies.

### 4.2. The Influence of Innate Biases on Bee Response to Tomato Floral Scent

The innate responses of insect pollinators to plant floral scent are not always consistent with their foraging behaviors on plants. Although the bumblebees performed better in foraging activity than the honeybees in the tomato greenhouses, they showed modest aversion to tomato floral scent (Figure 4A). In addition, we found that neither the honeybees nor the bumblebees extended their proboscises toward tomato floral scent innately (Figure 5). In natural ecosystems, both pollinators and non-pollinators impose selection on plant floral traits [48,49]. Plants use olfactory cues to attract bee pollinators. For example, bioassays have shown that naïve honeybees are attracted to apple floral scent [50]. However, many plants, including tomato, release repellent volatile organic compounds to defend against insect herbivores [51,52,53]. Plant defense against herbivores may show a trade-off with pollinator attraction [54]. In our study, both naïve honeybees and bumblebees exhibited aversion to tomato floral scent. Therefore, the trade-off between herbivore defense and pollinator attraction in tomato plants requires further investigation.

As a result of the adaptive strategies and coevolution between flowering plants and insects, pollinators have the ability to detect those plants with the highest food rewards, and plants have the ability to produce signals that attract their most effective pollinators [29]. In the ‘tomato-honeybee’ system, tomato is not a good food resource for honeybees, and honeybees are not efficient pollinators of tomato: pollen is the only food reward offered by tomato flowers, and only pollinators with ‘buzz pollination’ ability can obtain sufficient pollen from the poricidal anthers [8]. As they lack ‘buzz pollination’ ability, it is difficult for honeybees to obtain sufficient pollen grains from tomato flowers. Tomato floral scent may function as an indicator signal of poricidal anthers that honeybees potentially depend upon to identify ‘bad’ food resources. Further studies are needed to determine whether there are relationships between floral scent and anther traits in broader plant-insect systems.

### 4.3. The Influence of Prior Experience on Bee Response to Tomato Floral Scent

The innate responses of insect pollinators to scent may be overcome through the learning process [32]. Although the bees in this study showed an innate dislike for tomato floral scent, foraging experience or reward-learned experience changed their bias. Insect pollinators visit plants for food rewards and rely on floral scent to distinguish flowers with food rewards from deceptive ones [55,56]. When bees visit flowers, they acquire an ability to associate olfactory cues with food rewards [57,58]. It has been reported that the association of floral scent with a reward makes floral scent attractive to bees [35]. In our study, foraging experience in tomato greenhouses modified the preference of bumblebees for tomato floral scent. Although honeybees sampled from tomato greenhouses did not show any preference for tomato floral scent, when a sugar reward was associated with the floral scent via the PER procedure, honeybees developed a strong preference for tomato floral scent. Obtaining a food reward in prior experience strongly influences the scent preference of bees [59]. In the greenhouses in this study, honeybees collected limited amounts of tomato pollen grains and failed to develop a preference for tomato floral scent. However, once the honeybees learned to associate the tomato floral scent with the food rewards via the PER procedure, they were significantly attracted to the floral scent in the Y-tube. Farina et al.’s study showed that floral cues learned by members of a honeybee colony improved honeybee foraging activity on a target crop [60]. In our study, we investigated the influence of scent-learning experience on honeybees’ preference for tomato floral scent only in a Y-tube. Fieldwork is needed to determine whether scent learning improves honeybees’ foraging activity on tomato flowers in greenhouses.

Compared with bumblebees, honeybees took less time to associate tomato floral scent with sugar reward and exhibited a longer memory, thus showing better olfactory associative learning [61]. The bumblebee colonies in our study were fed commercial oilseed rape and apricot pollen in our study. Pollen from these intensively managed crops might be highly contaminated with chemical residues, such as pesticides. Further studies are needed to analyze the influences of pesticide residues in pollen paste on bumblebee learning behaviors. When searching for flowers or making foraging decisions, bumblebees depend more on individual foraging experience than on information from colony members, whereas honeybees tend to be more reliant upon gathering food reward information from their nest-mates [62]. Floral scent is one of the most important cues that honeybees depend upon to transmit food resource information among nest-mates [56]. When managing honeybee colonies to pollinate crops that have short flowering periods or are weakly attractive to honeybees, the use of floral scent learning might form an important part of a precision pollination strategy. In our study, due to the unsuccessful experience in gathering pollen from tomato flowers, tomato floral scent may be a deceptive odor signal for honeybees. Once a deceptive odor signal has developed and been transmitted in a honeybee colony, it is likely that fewer and fewer honeybee workers would forage on such flowers; such a scenario could explain why the foraging behavior of honeybees decreased significantly over the days after being placed in greenhouse.

## 5. Conclusions

Prior experience with food rewards can change the innate bias of bees toward floral scent. In our study, neither naïve bumblebees nor honeybees were attracted to tomato floral scent, but bumblebees could develop a preference for the floral scent following foraging experience. Given that honeybees rarely collect pollen from tomato flowers, we propose that the innate dislike of tomato floral scent and the unsuccessful food-collecting experience of honeybees explain their low foraging activity on tomato flowers. From the point of view of plant reproductive success, tomato floral scent might act as a repellent signal to deter ineffective pollinators, such as honeybees. However, in the present study, honeybees had better learning ability than the bumblebees and could develop a strong preference for the floral scent via the learning procedure. These results provide experimental support for the use of odors in pollination management. Fieldwork is needed to investigate whether the learning of floral scent influences honeybee foraging activity in tomato greenhouses and if so, how.

## References

[1] Food and Agricultural Organization of the United Nations (2018). Online Statistical Database. FAOSTAT. http://faostat.fao.org/default.aspx.

[2] Ministry of Agriculture and Rural Affairs of the People’s Repulic of China Summary of China National Agricultural Statistics. 2015 Statistical Division. http://www.moa.gov.cn/.

[3] Gong X., Wang S., Xu C., Zhang H., Ge J. (2020). Evaluation of several reference evapotranspiration models and determination of crop water requirement for tomato in a solar greenhouse. Hortscience.

[4] Wu G., Yang Q., Zhang Y., Fang H., Feng C., Zheng H. (2020). Energy and optical analysis of photovoltaic thermal integrated with rotary linear curved Fresnel lens inside a Chinese solar greenhouse. Energy.

[5] Zhang X., Lv J., Xie J., Yu J., Zhang J., Tang C., Li J., He Z., Wang C. (2020). Solar radiation allocation and spatial distribution in Chinese solar greenhouses: Model development and application. Energies.

[6] Free J.B. (1993). Insect Pollination of Crops.

[7] Bowers K.A.W. (1975). The pollination ecology of *Solanum rostratum* (Solanaceae). Am. J. Bot..

[8] Buchmann S.L., Cane J.H. (1989). Bees assess pollen returns while sonieating *Solanum* flowers. Oecologia.

[9] Arroyo-Correa B., Beattie C., Vallejo-Marín M. (2019). Bee and floral traits affect the characteristics of the vibrations experienced by flowers during buzz pollination. J. Exp. Biol..

[10] Velthuis H.H., van Doorn A. (2006). A century of advances in bumblebee domestication and the economic and environmental aspects of its commercialization for pollination. Apidologie.

[11] Hingston A.B., Marsden-Smedley J., Driscoll D.A., Corbett S., Fenton J., Anderson R., Plowman C., Mowling F., Jenkin M., Matsui K. (2002). Extent of invasion of Tasmanian native vegetation by the exotic bumblebee *Bombus terrestris* (Apoidea: Apidae). Austral Ecol..

[12] Matsumura C., Yokoyama J., Washitani I. (2004). Invasion status and potential ecological impacts of an invasive alien bumblebee, *Bombus terrestris* L. (Hymenoptera: Apidae) naturalized in southern Hokkaido, Japan. Glob. Environ. Res..

[13] Inoue M.N., Yokoyama J., Washitani I. (2008). Displacement of Japanese native bumblebees by the recently introduced *Bombus terrestris* (L.) (Hymenoptera: Apidae). J. Insect. Conserv..

[14] Morandin L.A., Laverty T.M., Kevan P.G. (2001). Bumble bee (Hymenoptera: Apidae) activity and pollination levels in commercial tomato greenhouses. J. Econ. Entomol..

[15] Cauich O., Quezada-Euán J.J.G., Macias-Macias J.O., Reyes-Oregel V., Medina-Peralta S., Parra-Tabla V. (2004). Behavior and pollination efficiency of *Nannotrigona perilampoides* (Hymenoptera: Meliponini) on greenhouse tomatoes (*Lycopersicon esculentum*) in subtropical México. J. Econ. Entomol..

[16] Hogendoorn K., Bartholomaeus F., Keller M.A. (2010). Chemical and sensory comparison of tomatoes pollinated by bees and by a pollination wand. J. Econ. Entomol..

[17] Huang J., An J. (2018). Species diversity, pollination application and strategy for conservation of the bumblebees of China. Biodiv. Sci..

[18] Zhang H., Zhou Z., Huang J., Yuan X., Ding G., An J. (2018). Queen traits and colony size of four bumblebee species of China. Insects Soc..

[19] Zhang H., Zhou Z., An J. (2019). Pollen release dynamics and daily patterns of pollen-collecting activity of honeybee *Apis mellifera* and bumblebee *Bombus lantschouensis* in solar greenhouse. Insects.

[20] Cane J.H., Mackenzie K., Schiffhauer D. (1993). Honey bees harvest pollen from the porose anthers of cranberries (*Vaccinium macrocarpon*) (Ericaceae). Am. Bee J..

[21] Cribb D., Hand D., Edmondson R. (1993). A comparative study of the effects of using the honeybee as a pollinating agent of glasshouse tomato. J. Hort. Sci..

[22] Higo H.A., Rice N.D., Winston M.L., Lewis B. (2004). Honey bee (Hymenoptera: Apidae) distribution and potential for supplementary pollination in commercial tomato greenhouses during winter. J. Econ. Entomol..

[23] Sabara H.A., Gillespie D.R., Elle E., Winston M.L. (2004). Influence of brood, vent screening, and time of year on honey bee (Hymenoptera: Apidae) pollination and fruit quality of greenhouse tomatoes. J. Econ. Entomol..

[24] Neiswander R.B. (1956). Pollination of greenhouse tomatoes by honey bees. J. Econ. Entomol..

[25] Banda H., Paxton R. (1991). Pollination of greenhouse tomatoes by bees. Acta Hortic..

[26] Bispo dos Santos S.A., Roselino A., Hrncir M., Bego L. (2009). Pollination of tomatoes by the stingless bee *Melipona quadrifasciata* and the honey bee *Apis mellifera* (Hymenoptera, Apidae). Genet. Mol. Res..

[27] Kessler D., Gase K., Baldwin I.T. (2008). Field experiments with transformed plants reveal the sense of floral scents. Science.

[28] Schiestl F.P., Johnson S.D. (2013). Pollinator-mediated evolution of floral signals. Trends. Ecol. Evol..

[29] Haber A.I., Sims J.W., Mescher M.C., De Moraes C.M., Carr D.E. (2018). A key floral scent component (β-*trans*-bergamotene) drives pollinator preferences independently of pollen rewards in seep monkeyflower. Funct. Ecol..

[30] Larue A.A., Raguso R.A., Junker R.R. (2016). Experimental manipulation of floral scent bouquets restructures flower-visitor interactions in the field. J. Anim. Ecol..

[31] Riffell J.A., Alarcon R., Abrell L., Davidowitz G., Bronstein J.L., Hildebrand J.G. (2008). Behavioral consequences of innate preferences and olfactory learning in hawkmoth-flower interactions. Proc. Natl. Acad. Sci. USA.

[32] Fujita M., Tanimura T. (2011). *Drosophila* evaluates and learns the nutritional value of sugars. Curr. Biol..

[33] Gong W.C., Chen G., Vereecken N.J., Dunn B.L., Ma Y.P., Sun W.B. (2015). Floral scent composition predicts bee pollination system in five butterfly bush (*Buddleja*, Scrophulariaceae) species. Plant Biol..

[34] Knauer A.C., Schiestl F.P. (2014). Bees use honest floral signals as indicators of reward when visiting flowers. Ecol. Lett..

[35] Sasso R., Iodice L., Woodcock C.M., Pickett J.A., Guerrieri E. (2009). Electrophysiological and behavioural responses of *Aphidius ervi* (Hymenoptera: Braconidae) to tomato plant volatiles. Chemoecology.

[36] Darshanee H.L.C., Ren H., Ahmed N., Zhang Z., Liu Y., Liu T. (2017). Volatile-mediated attraction of greenhouse whitefly *Trialeurodes vaporariorum* to tomato and eggplant. Front. Plant Sci..

[37] Paudel S., Lin P., Foolad M.R., Ali J.G., Rajotte E.G., Felton G.W. (2019). Induced plant defenses against herbivory in cultivated and wild tomato. J. Chem. Ecol..

[38] Morse A., Kevan P., Shipp L., Khosla S., McGarvey B. (2012). The impact of greenhouse tomato (Solanales: Solanaceae) floral volatiles on bumble bee (Hymenoptera: Apidae) pollination. Environ. Entomol..

[39] Vaissière B., Freitas B., Gemmill-Herren B. (2011). Protocol to Detect and Assess Pollination Deficits in Crops: A Handbook for Its Use.

[40] Huber F.K., Kaiser R., Sauter W., Schiestl F.P. (2005). Floral scent emission and pollinator attraction in two species of *Gymnadenia* (Orchidaceae). Oecologia.

[41] Suchet C., Dormont L., Schatz B., Giurfa M., Simon V., Raynaud C., Chave J. (2011). Floral scent variation in two *Antirrhinum majus* subspecies influences the choice of naïve bumblebees. Behav. Ecol. Sociobiol..

[42] Sommerlandt F.M.J., Roessler W., Spaethe J. (2014). Elemental and non-elemental olfactory learning using PER conditioning in the bumblebee, *Bombus terrestris*. Apidologie.

[43] Wang Z., Tan K. (2014). Comparative analysis of olfactory learning of *Apis cerana* and *Apis mellifera*. Apidologie.

[44] Delaplane K.S., Dag A., Danka R.G., Freitas B.M., Garibaldi L.A., Goodwin R.M., Hormaza J.I. (2013). Standard methods for pollination research with *Apis mellifera*. J. Apicult. Res..

[45] Gemeda T.K., Shao Y., Wu W., Yang H., Huang J., Wu J. (2017). Native honey bees outperform adventive honey bees in increasing *Pyrus bretschneideri* (Rosales: Rosaceae) pollination. J. Econ. Entomol..

[46] Sabara H.A., Winston M.L. (2003). Managing honey bees (Hymenoptera: Apidae) for greenhouse tomato pollination. J. Econ. Entomol..

[47] Dreller C., Page R.E., Fondrk M.K. (1999). Regulation of pollen foraging in honeybee colonies: Effects of young brood, stored pollen, and empty space. Behav. Ecol. Sociobiol..

[48] Strauss S.Y., Whittall J.B., Harder L.D., Barrett S.C.H. (2006). Non-pollinator agents of selection on floral traits. Ecology and Evolution of Flowers.

[49] Ramos S.E., Schiestl F.P. (2020). Evolution of floral fragrance is compromised by herbivory. Front. Ecol. Evol..

[50] Rachersberger M., Cordeiro G.D., Schäffler I., Dötterl S. (2019). Honeybee pollinators use visual and floral scent cues to find apple (*Malus domestica*) flowers. J. Agric. Food Chem..

[51] Ángeles L.Y.I., Martínez-Gallardo N.A., Ramírez-Romero R., López M.G., Sánchez-Hernández C., Délano-Frier J.P. (2012). Cross-kingdom effects of plant-plant signaling via volatile organic compounds emitted by tomato (*Solanum lycopersicum*) plants infested by the greenhouse whitefly (*Trialeurodes vaporariorum*). J. Chem. Ecol..

[52] Dawood M.H., Snyder J.C. (2020). The alcohol and epoxy alcohol of zingiberene, produced in trichomes of wild tomato, are more repellent to spider mites than zingiberene. Front. Plant Sci..

[53] Yang F., Zhang Q., Yao Q., Chen G., Tong H., Zhang J., Li C., Su Q., Zhang Y. (2020). Direct and indirect plant defenses induced by (Z)-3-hexenol in tomato against whitefly attack. J. Pest Sci..

[54] Lucas-Barbosa D., Sun P., Hakman A., van Beek T.A., van Loon J.J.A., Dicke M. (2016). Visual and odour cues: Plant responses to pollination and herbivory affect the behaviour of flower visitors. Funct. Ecol..

[55] Wright G.A., Schiestl F.P. (2009). The evolution of floral scent: The influence of olfactory learning by insect pollinators on the honest signaling of floral rewards. Funct. Ecol..

[56] Krug C., Cordeiro G.D., Schäffler I., Silva C.I., Oliveira R., Schlindwein C., Dötterl S., Alves-dos-Santos I. (2018). Nocturnal bee pollinators are attracted to Guarana flowers by their scents. Front. Plant Sci..

[57] Raguso R.A. (2008). Wake up and smell the roses: The ecology and evolution of floral scent. Annu. Rev. Ecol. Evol. Syst..

[58] Molet M., Chittka L., Raine N.E. (2009). How floral odours are learned inside the bumblebee (*Bombus terrestris*) nest. Sci. Nat..

[59] Russell A.L., Golden R.E., Leonard A.S., Papaj D.R. (2016). Bees learn preferences for plant species that offer only pollen as a reward. Behav. Ecol..

[60] Farina W.M., Arenas A., Díaz P.C., Martin C.S., Barcala M.C.E. (2020). Learning of a mimic odor within beehives improves pollination service efficiency in a commercial crop. Curr. Biol..

[61] Riveros A.J., Gronenberg W. (2009). Olfactory learning and memory in the bumblebee *Bombus occidentalis*. Sci. Nat..

[62] Townsend-Mehler J.M., Dyer F.C., Maida K. (2011). Deciding when to explore and when to persist: A comparison of honeybees and bumblebees in their response to downshifts in reward. Behav. Ecol. Sociobiol..

